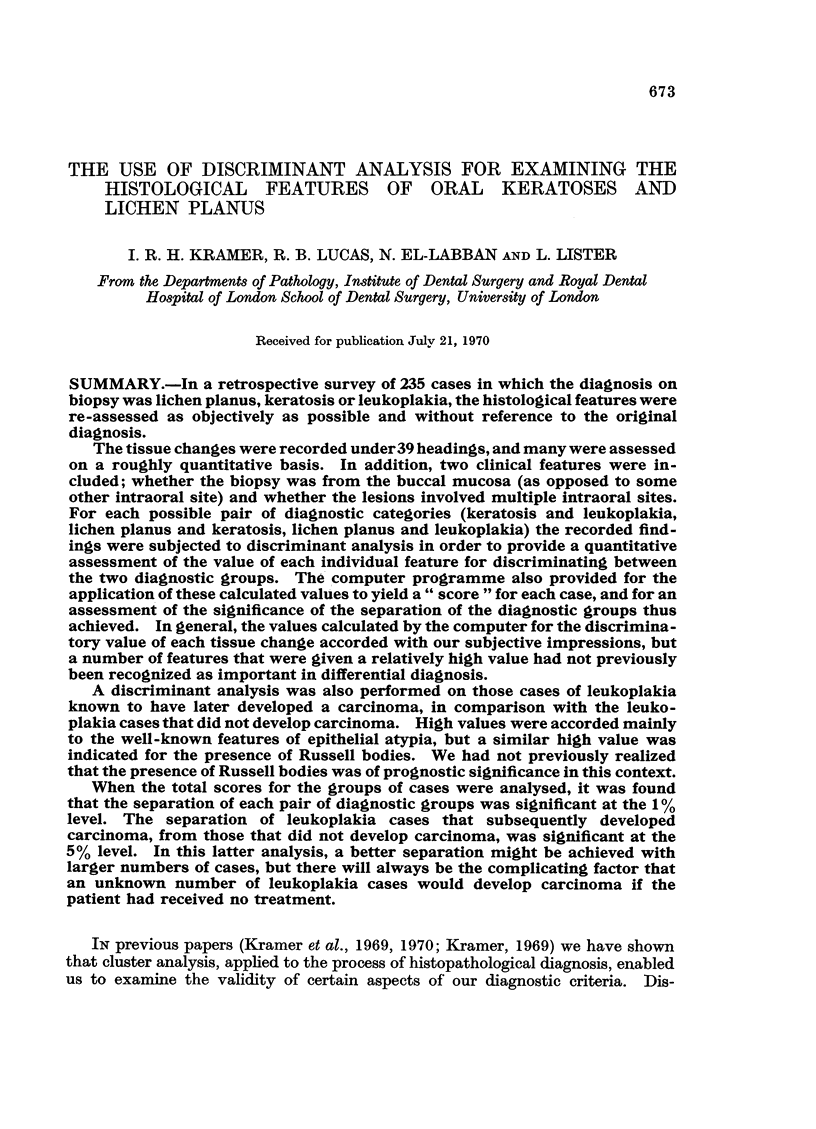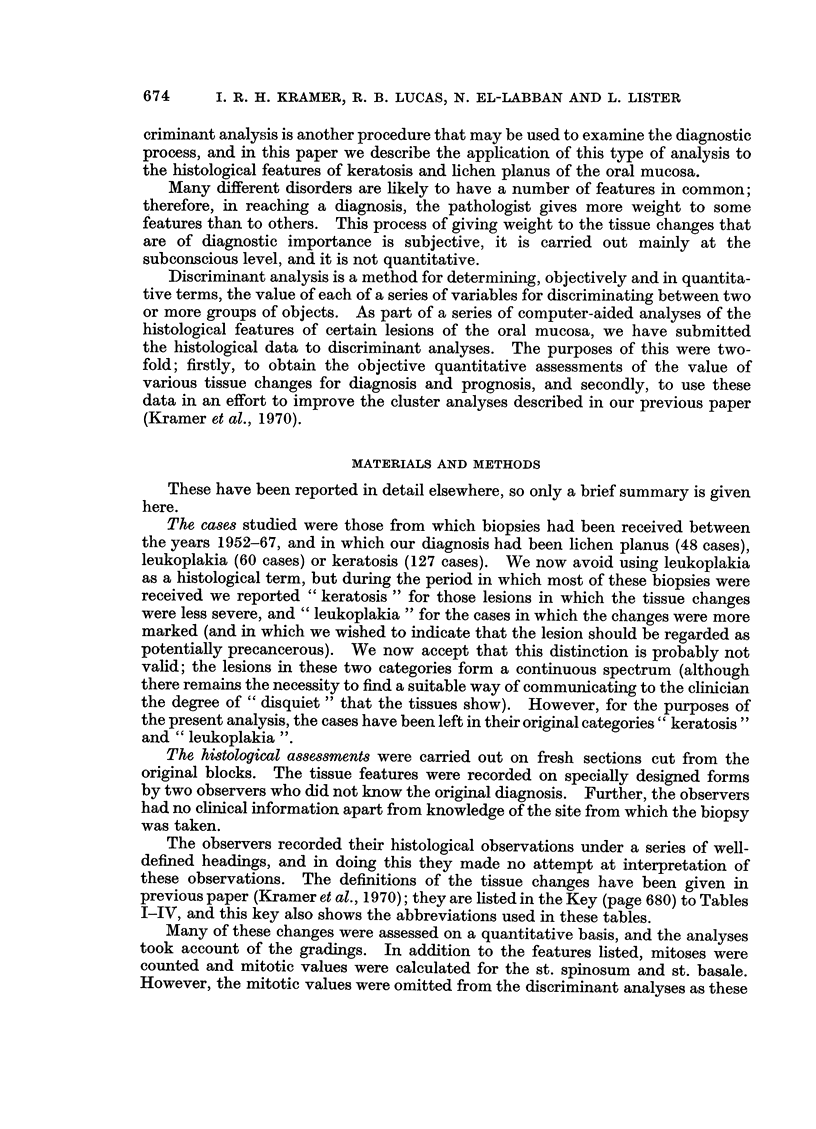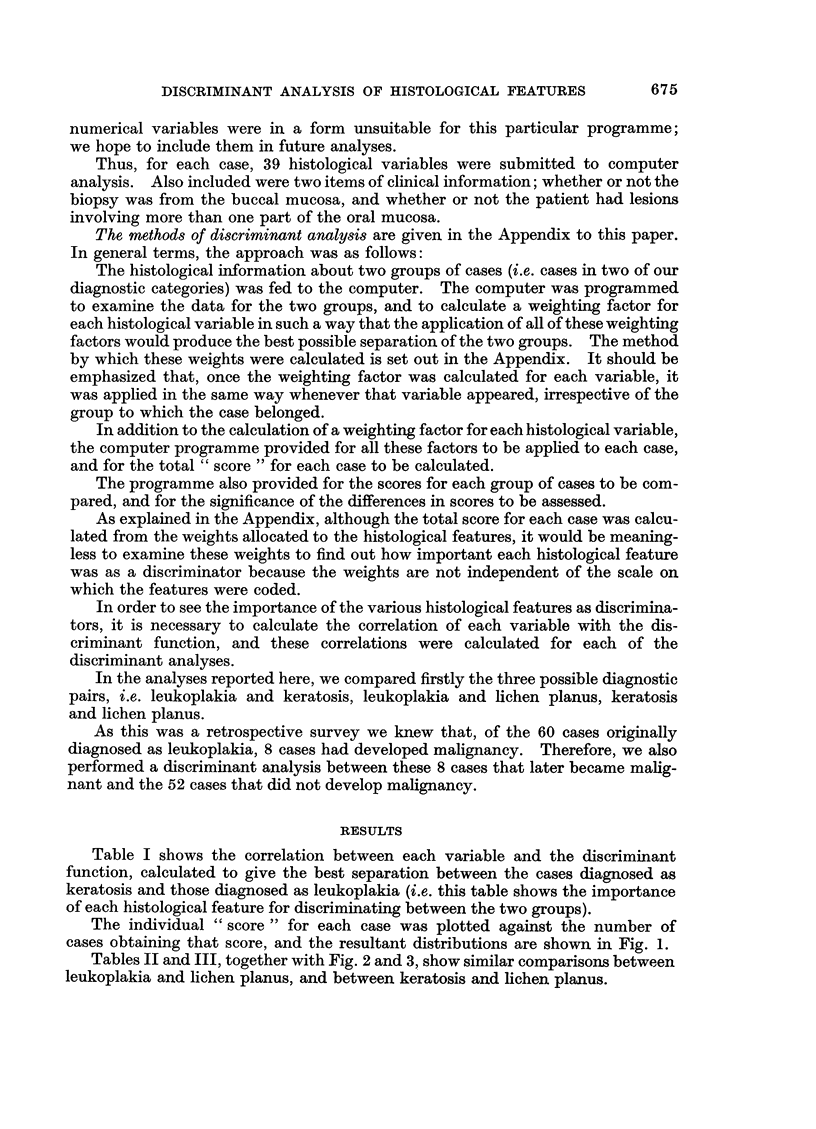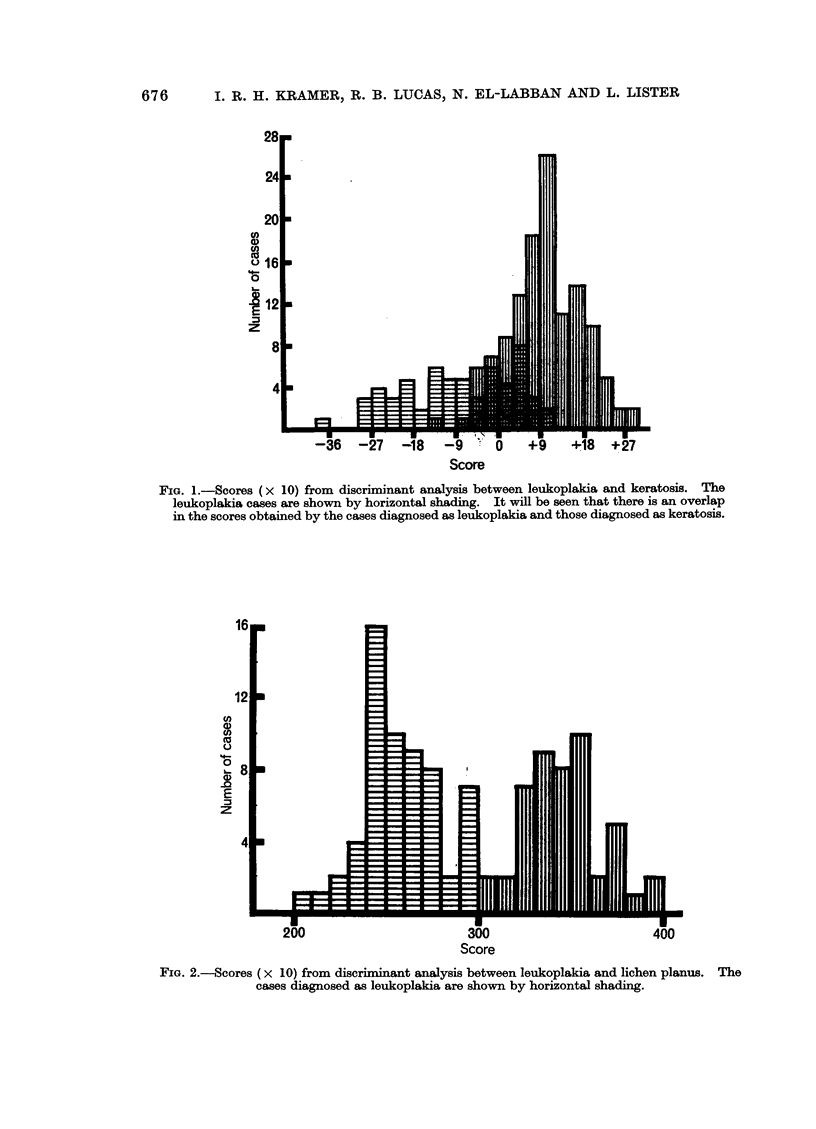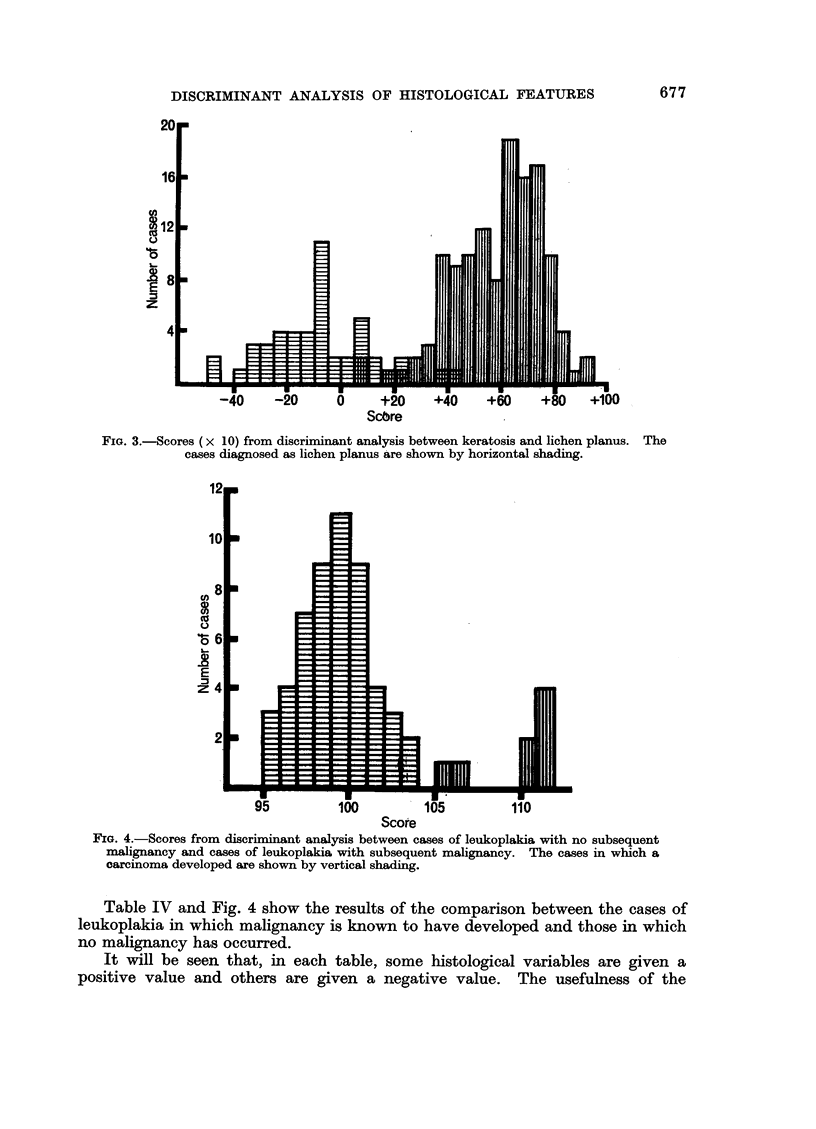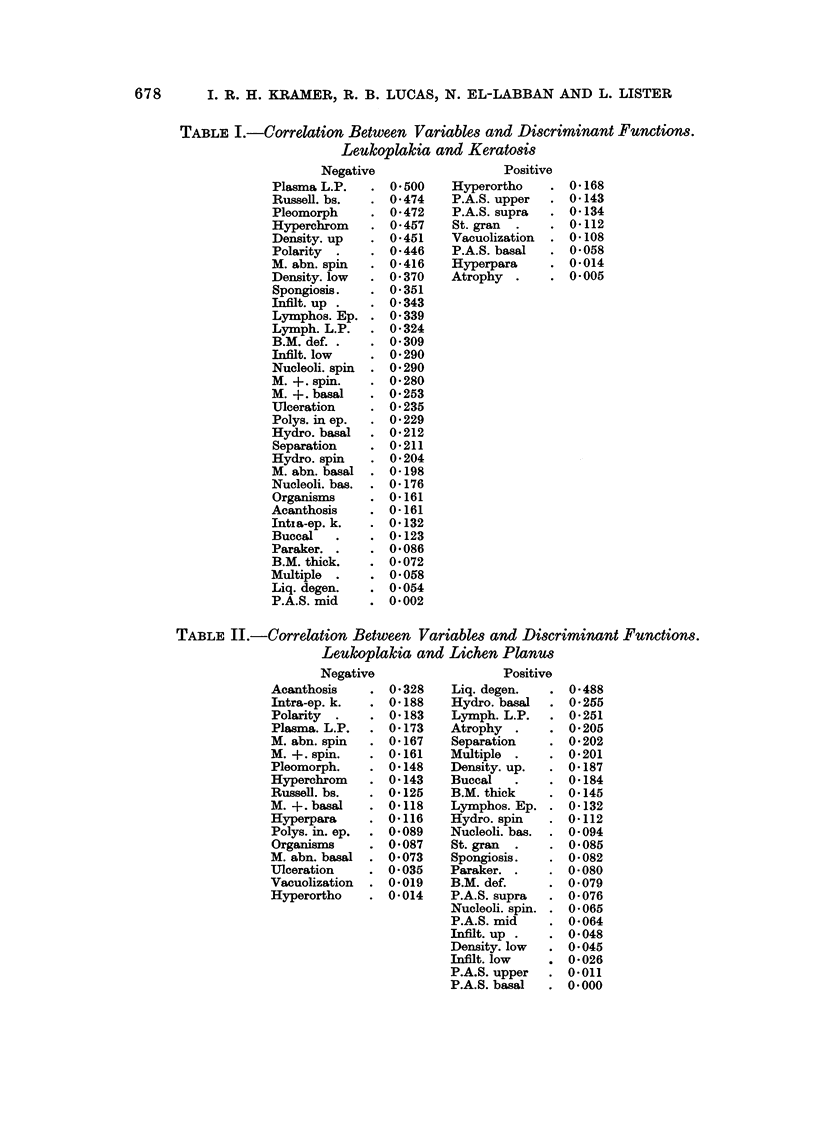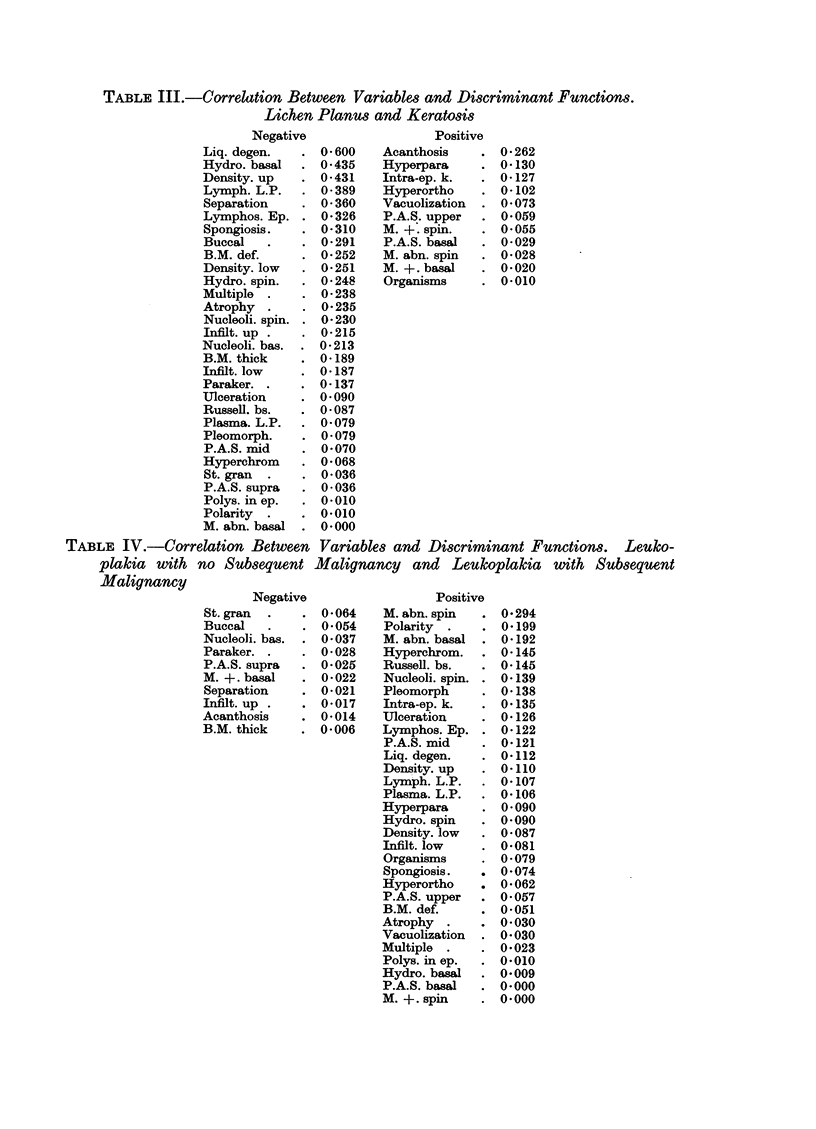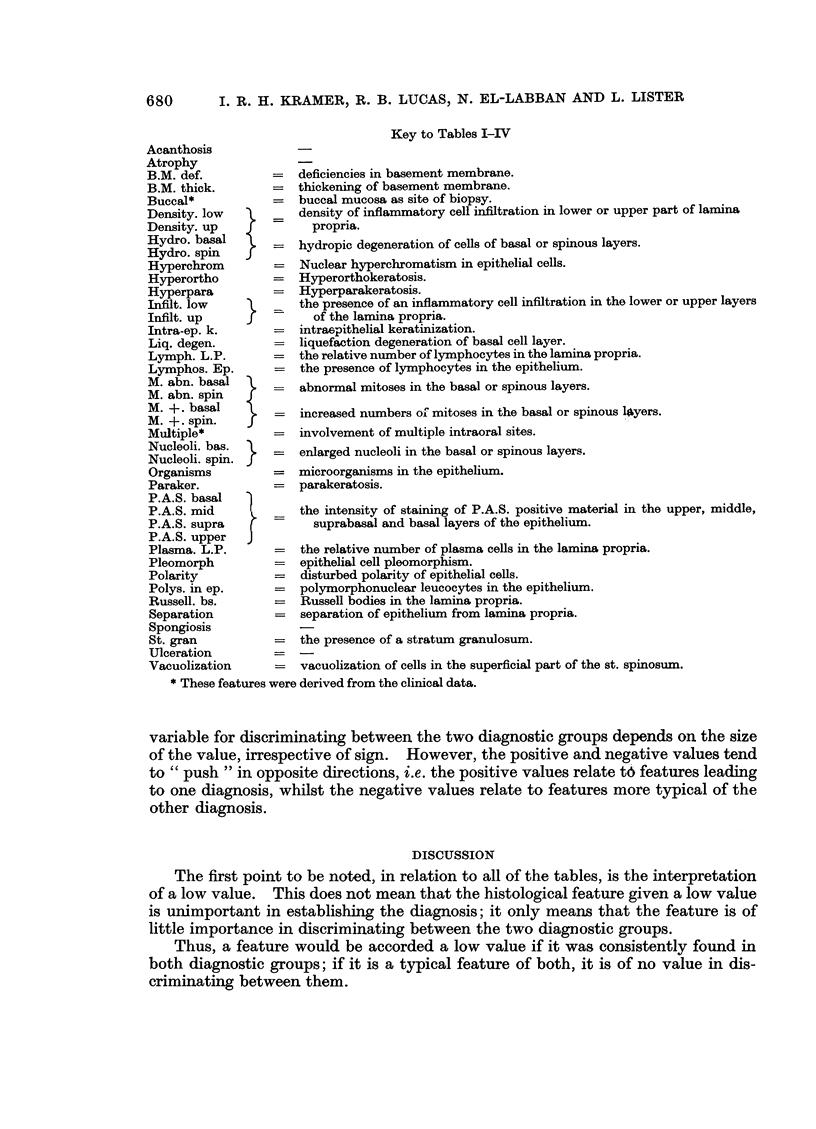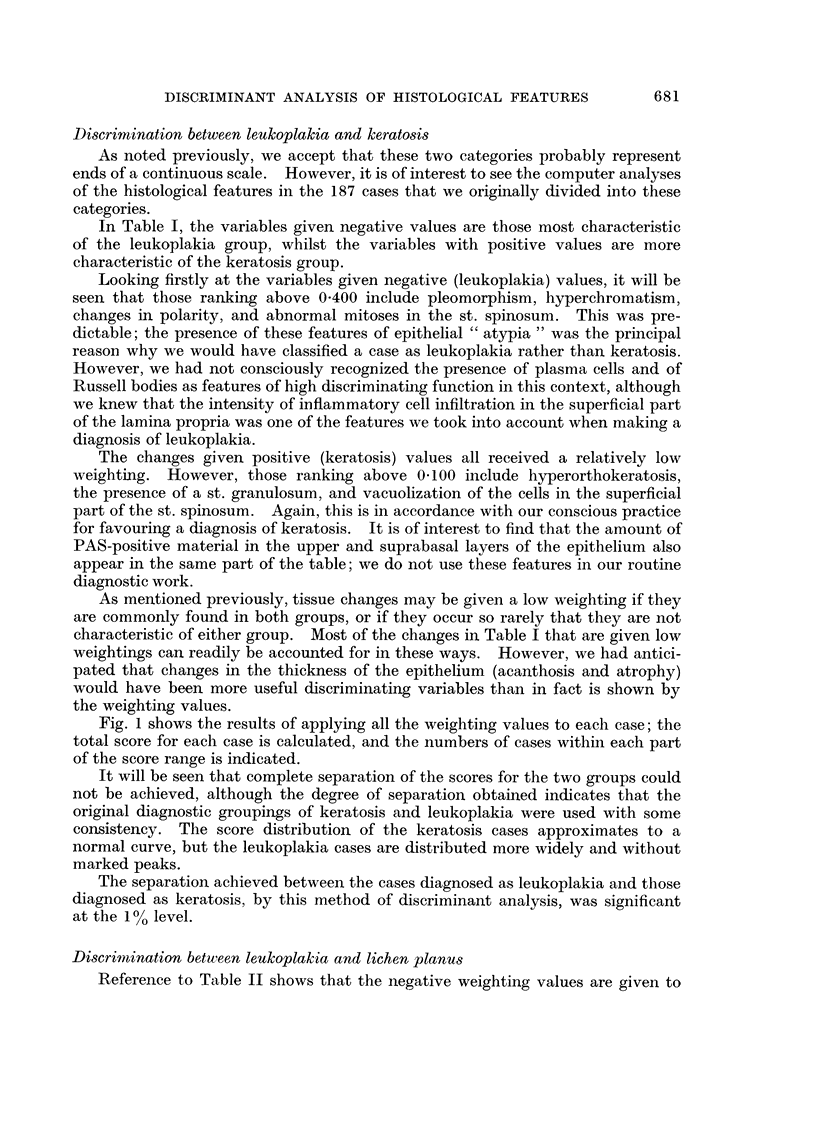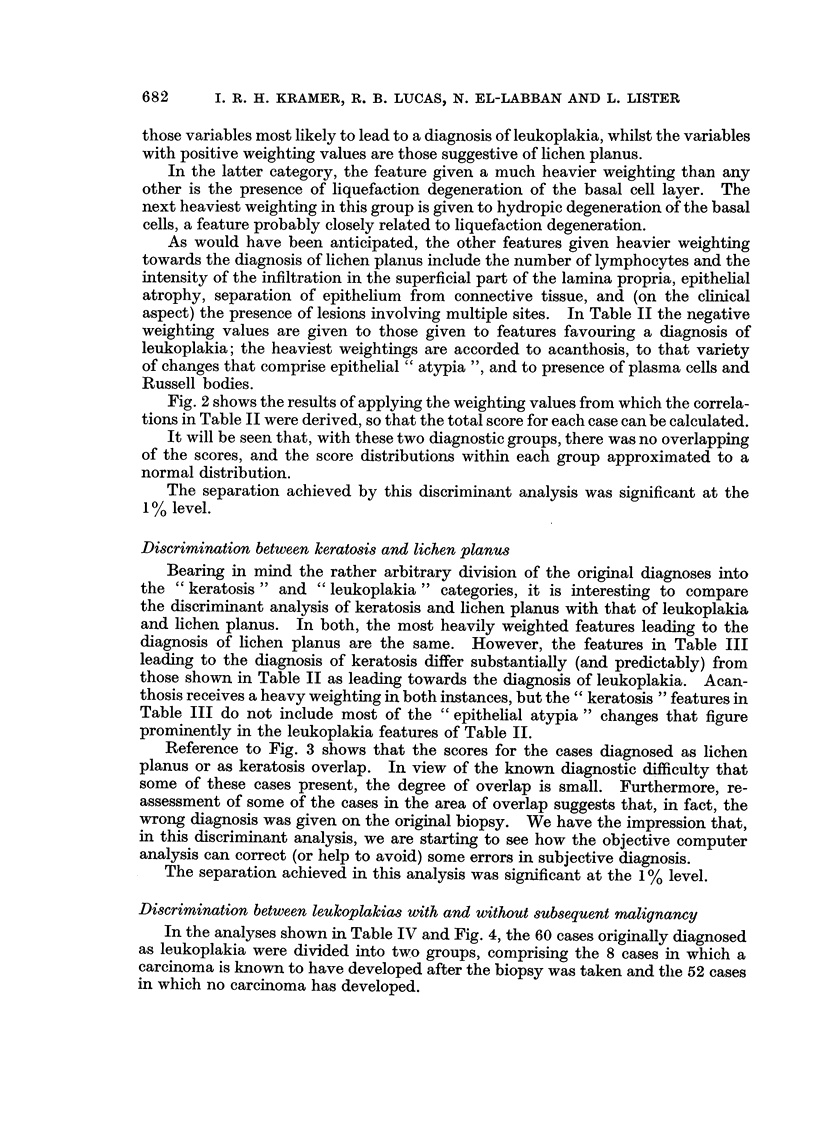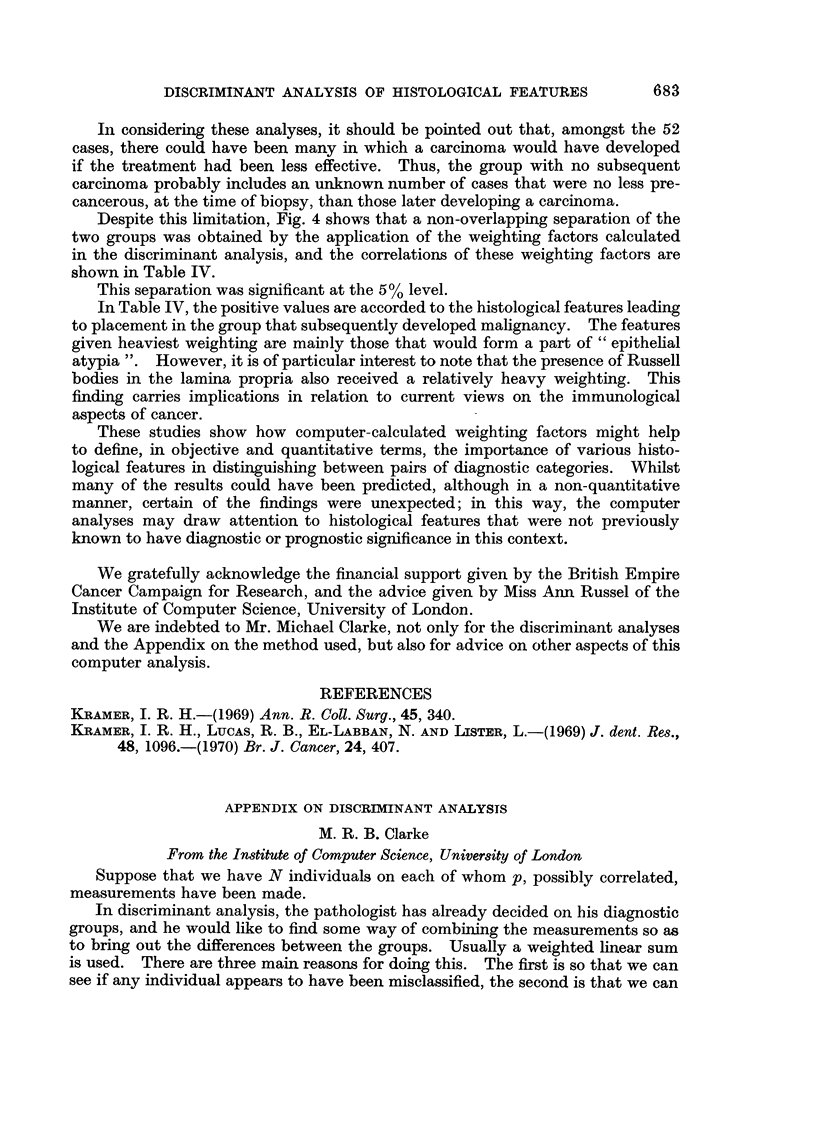# The Use of Discriminant Analysis for Examining the Histological Features of Oral Keratoses and Lichen Planus

**DOI:** 10.1038/bjc.1970.80

**Published:** 1970-12

**Authors:** I. R. H. Kramer, R. B. Lucas, N. El-Labban, L. Lister

## Abstract

In a retrospective survey of 235 cases in which the diagnosis on biopsy was lichen planus, keratosis or leukoplakia, the histological features were re-assessed as objectively as possible and without reference to the original diagnosis.

The tissue changes were recorded under 39 headings, and many were assessed on a roughly quantitative basis. In addition, two clinical features were included; whether the biopsy was from the buccal mucosa (as opposed to some other intraoral site) and whether the lesions involved multiple intraoral sites. For each possible pair of diagnostic categories (keratosis and leukoplakia, lichen planus and keratosis, lichen planus and leukoplakia) the recorded findings were subjected to discriminant analysis in order to provide a quantitative assessment of the value of each individual feature for discriminating between the two diagnostic groups. The computer programme also provided for the application of these calculated values to yield a “score” for each case, and for an assessment of the significance of the separation of the diagnostic groups thus achieved. In general, the values calculated by the computer for the discriminatory value of each tissue change accorded with our subjective impressions, but a number of features that were given a relatively high value had not previously been recognized as important in differential diagnosis

A discriminant analysis was also performed on those cases of leukoplakia known to have later developed a carcinoma, in comparison with the leukoplakia cases that did not develop carcinoma. High values were accorded mainly to the well-known features of epithelial atypia, but a similar high value was indicated for the presence of Russell bodies. we had not previously realized that the presence of Russell bodies was of prognostic significance in this context.

When the total scores for the groups of cases were analysed, it was found that the separation of each pair of diagnostic groups was significant at the 1% level. The separation of leukoplakia cases that subsequently developed carcinoma, from those that did not develop carcinoma, was significant at the 5% level. In this latter analysis, a better separation might be achieved with larger numbers of cases, but there will always be the complicating factor that an unknown number of leukoplakia cases would develop carcinoma if the patient had received no treatment.


### THE USE OF DISCRIMINANT ANALYSIS FOR EXAMINING THE HISTOLOGICAL FEATURES OF ORAL KERATOSES AND LICHEN PLANUS

I. R. H. KRAMER, R. B. LUCAS, N. EL-LABBAN AND L. LISTER

*From the Departments of Pathology, Institute of Dental Surgery and Royal Dental Hospital of London School of Dental Surgery, University of London*

Received for publication July 21, 1970

**SUMMARY.—In a retrospective survey of 235 cases in which the diagnosis on biopsy was lichen planus, keratosis or leukoplakia, the histological features were re-assessed as objectively as possible and without reference to the original diagnosis.**

**The tissue changes were recorded under 39 headings, and many were assessed on a roughly quantitative basis. In addition, two clinical features were included; whether the biopsy was from the buccal mucosa (as opposed to some other intraoral site) and whether the lesions involved multiple intraoral sites. For each possible pair of diagnostic categories (keratosis and leukoplakia, lichen planus and keratosis, lichen planus and leukoplakia) the recorded findings were subjected to discriminant analysis in order to provide a quantitative assessment of the value of each individual feature for discriminating between the two diagnostic groups. The computer programme also provided for the application of these calculated values to yield a " score " for each case, and for an assessment of the significance of the separation of the diagnostic groups thus achieved. In general, the values calculated by the computer for the discriminatory value of each tissue change accorded with our subjective impressions, but a number of features that were given a relatively high value had not previously been recognized as important in differential diagnosis.**

**A discriminant analysis was also performed on those cases of leukoplakia known to have later developed a carcinoma, in comparison with the leukoplakia cases that did not develop carcinoma. High values were accorded mainly to the well-known features of epithelial atypia, but a similar high value was indicated for the presence of Russell bodies. We had not previously realized that the presence of Russell bodies was of prognostic significance in this context.**

**When the total scores for the groups of cases were analysed, it was found that the separation of each pair of diagnostic groups was significant at the 1% level. The separation of leukoplakia cases that subsequently developed carcinoma, from those that did not develop carcinoma, was significant at the 5% level. In this latter analysis, a better separation might be achieved with larger numbers of cases, but there will always be the complicating factor that an unknown number of leukoplakia cases would develop carcinoma if the patient had received no treatment.**

IN previous papers (Kramer *et al.*, 1969, 1970; Kramer, 1969) we have shown that cluster analysis, applied to the process of histopathological diagnosis, enabled us to examine the validity of certain aspects of our diagnostic criteria. Discriminant analysis is another procedure that may be used to examine the diagnostic process, and in this paper we describe the application of this type of analysis to the histological features of keratosis and lichen planus of the oral mucosa.

Many different disorders are likely to have a number of features in common; therefore, in reaching a diagnosis, the pathologist gives more weight to some features than to others. This process of giving weight to the tissue changes that are of diagnostic importance is subjective, it is carried out mainly at the subconscious level, and it is not quantitative.

Discriminant analysis is a method for determining, objectively and in quantitative terms, the value of each of a series of variables for discriminating between two or more groups of objects. As part of a series of computer-aided analyses of the histological features of certain lesions of the oral mucosa, we have submitted the histological data to discriminant analyses. The purposes of this were two-fold; firstly, to obtain the objective quantitative assessments of the value of various tissue changes for diagnosis and prognosis, and secondly, to use these data in an effort to improve the cluster analyses described in our previous paper (Kramer *et al.*, 1970).

### MATERIALS AND METHODS

These have been reported in detail elsewhere, so only a brief summary is given here.

*The cases* studied were those from which biopsies had been received between the years 1952–67, and in which our diagnosis had been lichen planus (48 cases), leukoplakia (60 cases) or keratosis (127 cases). We now avoid using leukoplakia as a histological term, but during the period in which most of these biopsies were received we reported " keratosis " for those lesions in which the tissue changes were less severe, and " leukoplakia " for the cases in which the changes were more marked (and in which we wished to indicate that the lesion should be regarded as potentially precancerous). We now accept that this distinction is probably not valid; the lesions in these two categories form a continuous spectrum (although there remains the necessity to find a suitable way of communicating to the clinician the degree of " disquiet " that the tissues show). However, for the purposes of the present analysis, the cases have been left in their original categories " keratosis " and " leukoplakia ".

*The histological assessments* were carried out on fresh sections cut from the original blocks. The tissue features were recorded on specially designed forms by two observers who did not know the original diagnosis. Further, the observers had no clinical information apart from knowledge of the site from which the biopsy was taken.

The observers recorded their histological observations under a series of well-defined headings, and in doing this they made no attempt at interpretation of these observations. The definitions of the tissue changes have been given in previous paper (Kramer *et al.*, 1970); they are listed in the Key (page 680) to Tables I–IV, and this key also shows the abbreviations used in these tables.

Many of these changes were assessed on a quantitative basis, and the analyses took account of the gradings. In addition to the features listed, mitoses were counted and mitotic values were calculated for the st. spinosum and st. basale. However, the mitotic values were omitted from the discriminant analyses as these numerical variables were in a form unsuitable for this particular programme; we hope to include them in future analyses.

Thus, for each case, 39 histological variables were submitted to computer analysis. Also included were two items of clinical information; whether or not the biopsy was from the buccal mucosa, and whether or not the patient had lesions involving more than one part of the oral mucosa.

*The methods of discriminant analysis* are given in the Appendix to this paper. In general terms, the approach was as follows:

The histological information about two groups of cases (*i.e.* cases in two of our diagnostic categories) was fed to the computer. The computer was programmed to examine the data for the two groups, and to calculate a weighting factor for each histological variable in such a way that the application of all of these weighting factors would produce the best possible separation of the two groups. The method by which these weights were calculated is set out in the Appendix. It should be emphasized that, once the weighting factor was calculated for each variable, it was applied in the same way whenever that variable appeared, irrespective of the group to which the case belonged.

In addition to the calculation of a weighting factor for each histological variable, the computer programme provided for all these factors to be applied to each case, and for the total " score " for each case to be calculated.

The programme also provided for the scores for each group of cases to be compared, and for the significance of the differences in scores to be assessed.

As explained in the Appendix, although the total score for each case was calculated from the weights allocated to the histological features, it would be meaningless to examine these weights to find out how important each histological feature was as a discriminator because the weights are not independent of the scale on which the features were coded.

In order to see the importance of the various histological features as discriminators, it is necessary to calculate the correlation of each variable with the discriminant function, and these correlations were calculated for each of the discriminant analyses.

In the analyses reported here, we compared firstly the three possible diagnostic pairs, *i.e.* leukoplakia and keratosis, leukoplakia and lichen planus, keratosis and lichen planus.

As this was a retrospective survey we knew that, of the 60 cases originally diagnosed as leukoplakia, 8 cases had developed malignancy. Therefore, we also performed a discriminant analysis between these 8 cases that later became malignant and the 52 cases that did not develop malignancy.

### RESULTS

Table I shows the correlation between each variable and the discriminant function, calculated to give the best separation between the cases diagnosed as keratosis and those diagnosed as leukoplakia (*i.e.* this table shows the importance of each histological feature for discriminating between the two groups).

The individual " score " for each case was plotted against the number of cases obtaining that score, and the resultant distributions are shown in Fig. 1.

Tables II and III, together with Fig. 2 and 3, show similar comparisons between leukoplakia and lichen planus, and between keratosis and lichen planus.

FIG. 1.—Scores (× 10) from discriminant analysis between leukoplakia and keratosis. The leukoplakia cases are shown by horizontal shading. It will be seen that there is an overlap in the scores obtained by the cases diagnosed as leukoplakia and those diagnosed as keratosis.

FIG. 2.—Scores (× 10) from discriminant analysis between leukoplakia and lichen planus. The cases diagnosed as leukoplakia are shown by horizontal shading.

**Fig. 3.**—Scores (× 10) from discriminant analysis between keratosis and lichen planus. The cases diagnosed as lichen planus are shown by horizontal shading.

**Fig. 4.**—Scores from discriminant analysis between cases of leukoplakia with no subsequent malignancy and cases of leukoplakia with subsequent malignancy. The cases in which a carcinoma developed are shown by vertical shading.

Table IV and Fig. 4 show the results of the comparison between the cases of leukoplakia in which malignancy is known to have developed and those in which no malignancy has occurred.

It will be seen that, in each table, some histological variables are given a positive value and others are given a negative value. The usefulness of the

TABLE I.—*Correlation Between Variables and Discriminant Functions. Leukoplakia and Keratosis*

| Negative | | Positive | |
|---|---|---|---|
| Plasma L.P. | 0·500 | Hyperortho | 0·168 |
| Russell. bs. | 0·474 | P.A.S. upper | 0·143 |
| Pleomorph | 0·472 | P.A.S. supra | 0·134 |
| Hyperchrom | 0·457 | St. gran | 0·112 |
| Density. up | 0·451 | Vacuolization | 0·108 |
| Polarity | 0·446 | P.A.S. basal | 0·058 |
| M. abn. spin | 0·416 | Hyperpara | 0·014 |
| Density. low | 0·370 | Atrophy | 0·005 |
| Spongiosis | 0·351 | | |
| Infilt. up | 0·343 | | |
| Lymphos. Ep. | 0·339 | | |
| Lymph. L.P. | 0·324 | | |
| B.M. def. | 0·309 | | |
| Infilt. low | 0·290 | | |
| Nucleoli. spin | 0·290 | | |
| M. +. spin. | 0·280 | | |
| M. +. basal | 0·253 | | |
| Ulceration | 0·235 | | |
| Polys. in ep. | 0·229 | | |
| Hydro. basal | 0·212 | | |
| Separation | 0·211 | | |
| Hydro. spin | 0·204 | | |
| M. abn. basal | 0·198 | | |
| Nucleoli. bas. | 0·176 | | |
| Organisms | 0·161 | | |
| Acanthosis | 0·161 | | |
| Intra-ep. k. | 0·132 | | |
| Buccal | 0·123 | | |
| Paraker. | 0·086 | | |
| B.M. thick. | 0·072 | | |
| Multiple | 0·058 | | |
| Liq. degen. | 0·054 | | |
| P.A.S. mid | 0·002 | | |

TABLE II.—*Correlation Between Variables and Discriminant Functions. Leukoplakia and Lichen Planus*

| Negative | | Positive | |
|---|---|---|---|
| Acanthosis | 0·328 | Liq. degen. | 0·488 |
| Intra-ep. k. | 0·188 | Hydro. basal | 0·255 |
| Polarity | 0·183 | Lymph. L.P. | 0·251 |
| Plasma. L.P. | 0·173 | Atrophy | 0·205 |
| M. abn. spin | 0·167 | Separation | 0·202 |
| M. +. spin. | 0·161 | Multiple | 0·201 |
| Pleomorph. | 0·148 | Density. up. | 0·187 |
| Hyperchrom | 0·143 | Buccal | 0·184 |
| Russell. bs. | 0·125 | B.M. thick | 0·145 |
| M. +. basal | 0·118 | Lymphos. Ep. | 0·132 |
| Hyperpara | 0·116 | Hydro. spin | 0·112 |
| Polys. in. ep. | 0·089 | Nucleoli. bas. | 0·094 |
| Organisms | 0·087 | St. gran | 0·085 |
| M. abn. basal | 0·073 | Spongiosis. | 0·082 |
| Ulceration | 0·035 | Paraker. | 0·080 |
| Vacuolization | 0·019 | B.M. def. | 0·079 |
| Hyperortho | 0·014 | P.A.S. supra | 0·076 |
| | | Nucleoli. spin. | 0·065 |
| | | P.A.S. mid | 0·064 |
| | | Infilt. up | 0·048 |
| | | Density. low | 0·045 |
| | | Infilt. low | 0·026 |
| | | P.A.S. upper | 0·011 |
| | | P.A.S. basal | 0·000 |

TABLE III.—*Correlation Between Variables and Discriminant Functions. Lichen Planus and Keratosis*

| Negative | | Positive | |
|---|---|---|---|
| Liq. degen. | 0·600 | Acanthosis | 0·262 |
| Hydro. basal | 0·435 | Hyperpara | 0·130 |
| Density. up | 0·431 | Intra-ep. k. | 0·127 |
| Lymph. L.P. | 0·389 | Hyperortho | 0·102 |
| Separation | 0·360 | Vacuolization | 0·073 |
| Lymphos. Ep. | 0·326 | P.A.S. upper | 0·059 |
| Spongiosis. | 0·310 | M. +. spin. | 0·055 |
| Buccal | 0·291 | P.A.S. basal | 0·029 |
| B.M. def. | 0·252 | M. abn. spin | 0·028 |
| Density. low | 0·251 | M. +. basal | 0·020 |
| Hydro. spin. | 0·248 | Organisms | 0·010 |
| Multiple | 0·238 | | |
| Atrophy | 0·235 | | |
| Nucleoli. spin. | 0·230 | | |
| Infilt. up | 0·215 | | |
| Nucleoli. bas. | 0·213 | | |
| B.M. thick | 0·189 | | |
| Infilt. low | 0·187 | | |
| Paraker. | 0·137 | | |
| Ulceration | 0·090 | | |
| Russell. bs. | 0·087 | | |
| Plasma. L.P. | 0·079 | | |
| Pleomorph. | 0·079 | | |
| P.A.S. mid | 0·070 | | |
| Hyperchrom | 0·068 | | |
| St. gran | 0·036 | | |
| P.A.S. supra | 0·036 | | |
| Polys. in ep. | 0·010 | | |
| Polarity | 0·010 | | |
| M. abn. basal | 0·000 | | |

TABLE IV.—*Correlation Between Variables and Discriminant Functions. Leukoplakia with no Subsequent Malignancy and Leukoplakia with Subsequent Malignancy*

| Negative | | Positive | |
|---|---|---|---|
| St. gran | 0·064 | M. abn. spin | 0·294 |
| Buccal | 0·054 | Polarity | 0·199 |
| Nucleoli. bas. | 0·037 | M. abn. basal | 0·192 |
| Paraker. | 0·028 | Hyperchrom. | 0·145 |
| P.A.S. supra | 0·025 | Russell. bs. | 0·145 |
| M. +. basal | 0·022 | Nucleoli. spin. | 0·139 |
| Separation | 0·021 | Pleomorph | 0·138 |
| Infilt. up | 0·017 | Intra-ep. k. | 0·135 |
| Acanthosis | 0·014 | Ulceration | 0·126 |
| B.M. thick | 0·006 | Lymphos. Ep. | 0·122 |
| | | P.A.S. mid | 0·121 |
| | | Liq. degen. | 0·112 |
| | | Density. up | 0·110 |
| | | Lymph. L.P. | 0·107 |
| | | Plasma. L.P. | 0·106 |
| | | Hyperpara | 0·090 |
| | | Hydro. spin | 0·090 |
| | | Density. low | 0·087 |
| | | Infilt. low | 0·081 |
| | | Organisms | 0·079 |
| | | Spongiosis. | 0·074 |
| | | Hyperortho | 0·062 |
| | | P.A.S. upper | 0·057 |
| | | B.M. def. | 0·051 |
| | | Atrophy | 0·030 |
| | | Vacuolization | 0·030 |
| | | Multiple | 0·023 |
| | | Polys. in ep. | 0·010 |
| | | Hydro. basal | 0·009 |
| | | P.A.S. basal | 0·000 |
| | | M. +. spin | 0·000 |

Key to Tables I–IV

| | | |
|---|---|---|
| Acanthosis | | — |
| Atrophy | | — |
| B.M. def. | = | deficiencies in basement membrane. |
| B.M. thick. | = | thickening of basement membrane. |
| Buccal* | = | buccal mucosa as site of biopsy. |
| Density. low<br>Density. up | = | density of inflammatory cell infiltration in lower or upper part of lamina propria. |
| Hydro. basal<br>Hydro. spin | = | hydropic degeneration of cells of basal or spinous layers. |
| Hyperchrom | = | Nuclear hyperchromatism in epithelial cells. |
| Hyperortho | = | Hyperorthokeratosis. |
| Hyperpara | = | Hyperparakeratosis. |
| Infilt. low<br>Infilt. up | = | the presence of an inflammatory cell infiltration in the lower or upper layers of the lamina propria. |
| Intra-ep. k. | = | intraepithelial keratinization. |
| Liq. degen. | = | liquefaction degeneration of basal cell layer. |
| Lymph. L.P. | = | the relative number of lymphocytes in the lamina propria. |
| Lymphos. Ep. | = | the presence of lymphocytes in the epithelium. |
| M. abn. basal<br>M. abn. spin | = | abnormal mitoses in the basal or spinous layers. |
| M. +. basal<br>M. +. spin. | = | increased numbers of mitoses in the basal or spinous layers. |
| Multiple* | = | involvement of multiple intraoral sites. |
| Nucleoli. bas.<br>Nucleoli. spin. | = | enlarged nucleoli in the basal or spinous layers. |
| Organisms | = | microorganisms in the epithelium. |
| Paraker. | = | parakeratosis. |
| P.A.S. basal<br>P.A.S. mid<br>P.A.S. supra<br>P.A.S. upper | = | the intensity of staining of P.A.S. positive material in the upper, middle, suprabasal and basal layers of the epithelium. |
| Plasma. L.P. | = | the relative number of plasma cells in the lamina propria. |
| Pleomorph | = | epithelial cell pleomorphism. |
| Polarity | = | disturbed polarity of epithelial cells. |
| Polys. in ep. | = | polymorphonuclear leucocytes in the epithelium. |
| Russell. bs. | = | Russell bodies in the lamina propria. |
| Separation | = | separation of epithelium from lamina propria. |
| Spongiosis | | — |
| St. gran | = | the presence of a stratum granulosum. |
| Ulceration | = | — |
| Vacuolization | = | vacuolization of cells in the superficial part of the st. spinosum. |

* These features were derived from the clinical data.

variable for discriminating between the two diagnostic groups depends on the size of the value, irrespective of sign. However, the positive and negative values tend to " push " in opposite directions, *i.e.* the positive values relate to features leading to one diagnosis, whilst the negative values relate to features more typical of the other diagnosis.

### DISCUSSION

The first point to be noted, in relation to all of the tables, is the interpretation of a low value. This does not mean that the histological feature given a low value is unimportant in establishing the diagnosis; it only means that the feature is of little importance in discriminating between the two diagnostic groups.

Thus, a feature would be accorded a low value if it was consistently found in both diagnostic groups; if it is a typical feature of both, it is of no value in discriminating between them.

*Discrimination between leukoplakia and keratosis*

As noted previously, we accept that these two categories probably represent ends of a continuous scale. However, it is of interest to see the computer analyses of the histological features in the 187 cases that we originally divided into these categories.

In Table I, the variables given negative values are those most characteristic of the leukoplakia group, whilst the variables with positive values are more characteristic of the keratosis group.

Looking firstly at the variables given negative (leukoplakia) values, it will be seen that those ranking above 0·400 include pleomorphism, hyperchromatism, changes in polarity, and abnormal mitoses in the st. spinosum. This was predictable; the presence of these features of epithelial " atypia " was the principal reason why we would have classified a case as leukoplakia rather than keratosis. However, we had not consciously recognized the presence of plasma cells and of Russell bodies as features of high discriminating function in this context, although we knew that the intensity of inflammatory cell infiltration in the superficial part of the lamina propria was one of the features we took into account when making a diagnosis of leukoplakia.

The changes given positive (keratosis) values all received a relatively low weighting. However, those ranking above 0·100 include hyperorthokeratosis, the presence of a st. granulosum, and vacuolization of the cells in the superficial part of the st. spinosum. Again, this is in accordance with our conscious practice for favouring a diagnosis of keratosis. It is of interest to find that the amount of PAS-positive material in the upper and suprabasal layers of the epithelium also appear in the same part of the table; we do not use these features in our routine diagnostic work.

As mentioned previously, tissue changes may be given a low weighting if they are commonly found in both groups, or if they occur so rarely that they are not characteristic of either group. Most of the changes in Table I that are given low weightings can readily be accounted for in these ways. However, we had anticipated that changes in the thickness of the epithelium (acanthosis and atrophy) would have been more useful discriminating variables than in fact is shown by the weighting values.

Fig. 1 shows the results of applying all the weighting values to each case; the total score for each case is calculated, and the numbers of cases within each part of the score range is indicated.

It will be seen that complete separation of the scores for the two groups could not be achieved, although the degree of separation obtained indicates that the original diagnostic groupings of keratosis and leukoplakia were used with some consistency. The score distribution of the keratosis cases approximates to a normal curve, but the leukoplakia cases are distributed more widely and without marked peaks.

The separation achieved between the cases diagnosed as leukoplakia and those diagnosed as keratosis, by this method of discriminant analysis, was significant at the 1% level.

*Discrimination between leukoplakia and lichen planus*

Reference to Table II shows that the negative weighting values are given to those variables most likely to lead to a diagnosis of leukoplakia, whilst the variables with positive weighting values are those suggestive of lichen planus.

In the latter category, the feature given a much heavier weighting than any other is the presence of liquefaction degeneration of the basal cell layer. The next heaviest weighting in this group is given to hydropic degeneration of the basal cells, a feature probably closely related to liquefaction degeneration.

As would have been anticipated, the other features given heavier weighting towards the diagnosis of lichen planus include the number of lymphocytes and the intensity of the infiltration in the superficial part of the lamina propria, epithelial atrophy, separation of epithelium from connective tissue, and (on the clinical aspect) the presence of lesions involving multiple sites. In Table II the negative weighting values are given to those given to features favouring a diagnosis of leukoplakia; the heaviest weightings are accorded to acanthosis, to that variety of changes that comprise epithelial " atypia ", and to presence of plasma cells and Russell bodies.

Fig. 2 shows the results of applying the weighting values from which the correlations in Table II were derived, so that the total score for each case can be calculated.

It will be seen that, with these two diagnostic groups, there was no overlapping of the scores, and the score distributions within each group approximated to a normal distribution.

The separation achieved by this discriminant analysis was significant at the 1% level.

*Discrimination between keratosis and lichen planus*

Bearing in mind the rather arbitrary division of the original diagnoses into the " keratosis " and " leukoplakia " categories, it is interesting to compare the discriminant analysis of keratosis and lichen planus with that of leukoplakia and lichen planus. In both, the most heavily weighted features leading to the diagnosis of lichen planus are the same. However, the features in Table III leading to the diagnosis of keratosis differ substantially (and predictably) from those shown in Table II as leading towards the diagnosis of leukoplakia. Acanthosis receives a heavy weighting in both instances, but the " keratosis " features in Table III do not include most of the " epithelial atypia " changes that figure prominently in the leukoplakia features of Table II.

Reference to Fig. 3 shows that the scores for the cases diagnosed as lichen planus or as keratosis overlap. In view of the known diagnostic difficulty that some of these cases present, the degree of overlap is small. Furthermore, reassessment of some of the cases in the area of overlap suggests that, in fact, the wrong diagnosis was given on the original biopsy. We have the impression that, in this discriminant analysis, we are starting to see how the objective computer analysis can correct (or help to avoid) some errors in subjective diagnosis.

The separation achieved in this analysis was significant at the 1% level.

*Discrimination between leukoplakias with and without subsequent malignancy*

In the analyses shown in Table IV and Fig. 4, the 60 cases originally diagnosed as leukoplakia were divided into two groups, comprising the 8 cases in which a carcinoma is known to have developed after the biopsy was taken and the 52 cases in which no carcinoma has developed.

In considering these analyses, it should be pointed out that, amongst the 52 cases, there could have been many in which a carcinoma would have developed if the treatment had been less effective. Thus, the group with no subsequent carcinoma probably includes an unknown number of cases that were no less precancerous, at the time of biopsy, than those later developing a carcinoma.

Despite this limitation, Fig. 4 shows that a non-overlapping separation of the two groups was obtained by the application of the weighting factors calculated in the discriminant analysis, and the correlations of these weighting factors are shown in Table IV.

This separation was significant at the 5% level.

In Table IV, the positive values are accorded to the histological features leading to placement in the group that subsequently developed malignancy. The features given heaviest weighting are mainly those that would form a part of "epithelial atypia". However, it is of particular interest to note that the presence of Russell bodies in the lamina propria also received a relatively heavy weighting. This finding carries implications in relation to current views on the immunological aspects of cancer.

These studies show how computer-calculated weighting factors might help to define, in objective and quantitative terms, the importance of various histological features in distinguishing between pairs of diagnostic categories. Whilst many of the results could have been predicted, although in a non-quantitative manner, certain of the findings were unexpected; in this way, the computer analyses may draw attention to histological features that were not previously known to have diagnostic or prognostic significance in this context.

We gratefully acknowledge the financial support given by the British Empire Cancer Campaign for Research, and the advice given by Miss Ann Russel of the Institute of Computer Science, University of London.

We are indebted to Mr. Michael Clarke, not only for the discriminant analyses and the Appendix on the method used, but also for advice on other aspects of this computer analysis.

### REFERENCES

KRAMER, I. R. H.—(1969) *Ann. R. Coll. Surg.*, **45**, 340.

KRAMER, I. R. H., LUCAS, R. B., EL-LABBAN, N. AND LISTER, L.—(1969) *J. dent. Res.*, **48**, 1096.—(1970) *Br. J. Cancer*, **24**, 407.